# Motor dysfunction as a primary symptom predicts poor outcome: multicenter study of glioma symptoms

**DOI:** 10.3389/fonc.2023.1305725

**Published:** 2024-01-04

**Authors:** Tomi Kivioja, Jussi P. Posti, Jussi Sipilä, Minna Rauhala, Janek Frantzén, Maria Gardberg, Melissa Rahi, Kirsi Rautajoki, Matti Nykter, Ville Vuorinen, Kristiina Nordfors, Hannu Haapasalo, Joonas Haapasalo

**Affiliations:** ^1^ Faculty of Medicine and Health Technology, University of Tampere, Tampere, Finland; ^2^ Neurocenter, Department of Neurosurgery and Turku Brain Injury Center, Turku University Hospital and University of Turku, Turku, Finland; ^3^ Department of Neurology, Siun Sote, North Karelia Central Hospital, Joensuu, Finland; ^4^ Clinical Neurosciences, University of Turku, Turku, Finland; ^5^ Department of Neurosurgery, Tampere University Hospital and Tampere University, Tampere, Finland; ^6^ Department of Neurosurgery, Turku University Hospital and University of Turku, Turku, Finland; ^7^ Turku University Hospital, Tyks Laboratories, Pathology and Institute of Biomedicine, University of Turku, Turku, Finland; ^8^ Prostate Cancer Research Center, Faculty of Medicine and Health Technology, Tampere University and Tays Cancer Center, Tampere, Finland; ^9^ Faculty of Medicine and Health Technology, Tampere University and Tays Cancer Center, Tampere, Finland; ^10^ Department of Pediatrics, Tampere University Hospital, Tampere, Finland; ^11^ Fimlab Laboratories Ltd., Tampere University Hospital, Tampere, Finland

**Keywords:** glioma, brain neoplasm, symptoms, prognosis, motor dysfunction, epilepsy, cognitive disorder, headache

## Abstract

**Background and objectives:**

The objectives of this study were to investigate the prognostic value of primary symptoms and leading symptoms in adult patients with diffuse infiltrating glioma and to provide a clinical perspective for evaluating survival.

**Methods:**

This study included a retrospective cohort from two tertiary university hospitals (*n* = 604, 2006–2013, Tampere University Hospital and Turku University Hospital) and a prospective cohort (*n* = 156, 2014–2018, Tampere University Hospital). Preoperative symptoms were divided into primary and leading symptoms. Results were validated with the newer WHO 2021 classification criteria.

**Results:**

The most common primary symptoms were epileptic seizure (30.8% retrospective, 28.2% prospective), cognitive disorder (13.2% retrospective, 16.0% prospective), headache (8.6% retrospective, 12.8% prospective), and motor paresis (7.0% retrospective, 7.1% prospective). Symptoms that predicted better survival were epileptic seizure and visual or other sense-affecting symptom in the retrospective cohort and epileptic seizure and headache in the prospective cohort. Predictors of poor survival were cognitive disorder, motor dysfunction, sensory symptom, tumor hemorrhage, speech disorder and dizziness in the retrospective cohort and cognitive disorder, motor dysfunction, sensory symptom, and dizziness in the prospective cohort. Motor dysfunction served as an independent predictor of survival in a multivariate model (OR = 1.636).

**Conclusion:**

Primary and leading symptoms in diffuse gliomas are associated with prognoses in retrospective and prospective settings. Motor paresis was an independent prognostic factor for poor survival in multivariate analysis for grade 2-4 diffuse gliomas, especially in glioblastomas.

## 1 Introduction

Diffuse gliomas are brain tumors that are characterized by their resemblance to glial cells and include, for example, astrocytomas and oligodendrogliomas. Recently, the WHO classification of brain tumors introduced genetic and molecular information to diagnostics; *IDH* mutation status categorizes astrocytomas in their own subclasses. Conversely, both *IDH* mutation and 1p19q co-deletion characterize oligodendrogliomas (1). Grade 4 gliomas were divided into *IDH* mutated astrocytomas and *IDH* wildtype glioblastomas (GBM).

Brain tumors present with various symptoms, which can make the diagnosis difficult. Depending on the location and the ability to infiltrate brain tissue, symptoms can vary widely (2). In addition, it is common to present with multiple symptoms before any examination. Some symptoms are more noticeable and might lead to patients to seek help sooner. However, many symptoms (e.g. cognitive disorder or headache) may progress slower, so the diagnosis may be delayed (3, 4). Preoperative symptoms can be clustered into primary symptoms (PS) and leading symptoms (LS). PS are the first symptoms manifested by the tumor. However, the PS are not always the reason the patient seeks medical attention. LS are those that most likely lead to examination.

According to earlier studies, epileptic seizure is the most common PS in patients with diffuse glioma (2, 5). Though more prevalent in grades 2 and 3, seizures are still common in grade 4 gliomas. Cognitive disorder, headache, and motor paresis are common presenting symptoms in high-grade gliomas (HGG) (2–4). Certain patient characteristics (e.g. age and sex) might be associated with these symptoms. The molecular properties of glioma have been studied in relation to presenting symptoms to some extent. However, the focus has largely been on the associations with epilepsy (6). Posti et al (7) reported that cognitive disorder was associated with GBM especially in older patients.

Survival after glioma diagnosis is influenced by patient age, *IDH* mutation status, extent of surgery, location of the tumor, and perioperative Karnofsky performance score (8–14). Preoperative epileptic seizure is associated with better survival in patients with diffuse glioma (15–17), whereas cognitive disorder has been associated with worse survival, although not as an independent factor (18). Preoperative neurologic deficit has been associated with worse survival in low-grade gliomas (19). However, literature on this subject remains scarce. Furthermore, prognosis remains poor, especially with the most malignant subtype, GBM. An individual treatment plan must be evaluated in every patient to optimize the prognosis and to maintain the best possible neurological status. Thus, more data are needed on the determinants of survival.

No large studies focus on the PS in gliomas as outcome predictors. Moreover, to our knowledge, within the era of current molecular diagnostics, the prognostic capability of different symptoms has not been evaluated. Here, we aim to study the PS and LS in patients with glioma in a retrospective, two-center cohort (group 1) and a prospective, single-center cohort (group 2).

## 2 Materials and methods

### 2.1 Patient cohorts

A two-center retrospective cohort study was conducted at Tampere University Hospital and Turku University Hospital, both in Finland. These cohorts formed group 1 of this study. The group consisted of consecutive patients who had a primary resection or biopsy on grade 2–4 glioma during 2006**–**2013. Criteria for the cohort were a diagnosed WHO grade 2–4 diffuse astrocytoma or oligodendroglioma by MRI and/or CT and histological analysis. Only adult patients age 18 years or older at the time of the primary diagnosis were included. A total of 604 patients met the selection criteria. PS and LS were evaluated retrospectively from patient archives.

A prospective cohort was recruited from Tampere University Hospital during 2014–2018 and formed group 2 of this study. A patient history was obtained, and clinical information was recorded on the patients’ charts. A total of 250 patients with intracranial tumors were recruited for the prospective study. All recruited patients had undergone neurosurgical resection or biopsy. All patients received diagnoses according to the WHO 2016 classification of brain tumors (1). The inclusion criteria matched the retrospective criteria. From the 250 patients, 156 met the inclusion criteria. Only adult patients with diffuse glioma and primary operations were selected for the analyses. The data were collected with a detailed history-taking and a neurological status review before and after the operation. Patient characteristics are shown in **Table 1**.

**Table 1:** Patient characteristics in the retrospective cohorts (group 1) and the prospective cohort (group 2).

Patient characteristics	Group 1	Group 2
**No. of patients**	604	156
Sex		
**Male**	349 (57.8%)	87 (55.8%)
**Female**	255 (42.2%)	69 (44.2%)
Age, y		
**Median**	61	61
**Minimum**	20	21
**Maximum**	85	82
Grade		
**2**	108 (17.9%)	23 (14.7%)
***IDH* mutant/*IDH* wildtype/unknown**	42/26/40	13/2/8
**1p19q co-deleted/no co-deletion/unknown**	25/23/60	8/6/9
**3**	74 (12.3%)	28 (17.9%)
***IDH* mutant/*IDH* wildtype/unknown**	19/21/34	16/12/0
**1p19q co-deleted/no co-deletion/unknown**	2/28/44	9/5/14
**4**	422 (69.9%)	105 (67.3%)
***IDH* mutant/*IDH* wildtype/unknown**	22/248/152	4/98/3
Follow-up		
**No. of survivors at the end of follow-up**	69	87
**Median follow-up duration for survivors, days**	1896	263
**Median time from operation to death, days**	288	321

After the study was conducted, a new WHO criterion for glioma classification was published. The molecular data needed for the new classification was not present in all the gliomas, especially in group 1 for its retrospective nature. In this group the *IDH* status and 1p19q codeletion is known for total of 384 patients. Group 2 represents the newer era as a prospective group, where most gliomas were analyzed for *IDH* mutation and 1p19q codeletion. In fact, 145 of these has this data available. There was no availability for TERT promoter, MGMT, CDKN2A/B or chromosomal 7/10 deletion analyses. With this aforementioned data available, we did additional analyses for astrocytomas that had *IDH* data available and separated *IDH* wildtype glioblastomas from grade 4, *IDH* mutant astrocytomas. In addition, former grade 2-3 oligoastrocytomas were divided into astrocytomas and oligodendrogliomas. After this, oligodendroglial tumors were removed from the analyses. Patients who had a surgical resection were included in these updated analyses.

### 2.2 Data collection

Data were analyzed from these two aforementioned groups: group 1, which represented the retrospective two-center cohorts, and group 2, which represented the prospective single-center cohort (**Table 1**). Both groups were divided into two subgroups: subgroup A included WHO 2016 grade 2–3 gliomas, and subgroup B included grade 4 gliomas.

Symptoms were considered PS only if they were clearly first-onset symptoms. Patients with multiple first-onset symptoms or slowly progressing symptoms that could not be evaluated in a respective order were unable to be determined. If a patient presented with multiple symptoms, the LS was considered the one that most likely led to additional examinations. Patients presenting with only one symptom had the same PS and LS.

Symptoms were characterized specifically and then grouped into nine major symptom classes: epileptic seizure, cognitive disorder, motor dysfunction, headache, sensory symptom, visual or other sense-impairing symptom, tumor hemorrhage, speech disorder, and dizziness. Motor disorder included any paresis or paralysis logical to the anatomical localization of the tumor. Motor and sensory types of aphasia and dysarthria were classified as speech disorders. Cognitive disorders include loss of memory, weakening of everyday performance, inability to cooperate, and other problems with cognitive abilities. The symptoms were collected similarly as previously described (7). These included also hemorrhage, which, in fact, is not a symptom but rather a finding.

### 2.3 Neuropathological analysis

The brain tumor samples were h istologically diagnosed according to the WHO 2007 Classification of CNS Tumors in the retrospective cohorts (20) and according to the WHO 2016 Classification of CNS Tumors (1) in the prospective cohort. In the prospective cohort and in part of the retrospective samples, *IDH1* mutation status was determined using immunohistochemistry to identify the mutant R132H isocitrate dehydrogenase 1 (*IDH*1) protein (21, 22). Fluorescence *in situ* hybridization (FISH) assessment of the chromosomal arms 1p 19q was performed in oligodendroglial tumors (23).

### 2.4 Statistical analysis

All statistical analyses were conducted using SPSS 20.0 software (IBM, Armonk, NY). Prognostic values of PS and LS were analyzed by Kaplan-Meier curves with log-rank tests. Cox multivariate regression analysis was used to assess the independent value of the prognostic factors in univariate analysis. Covariates used for the Cox forward stepwise regression model were PS, LS, age groups, tumor grade, *IDH* mutation status, and preoperative Karnofsky performance scale. Age groups were divided into four equal-sized groups; the minimum age was 20 years, and the maximum age was 85 years.

Cause of death data were retrieved from Statistics Finland, an institution that collects vital status data for all Finnish citizens and enters it into the Finnish Causes of Death Register. The cause of death data contains information about primary and ancillary causes of death. We used these data, together with the date of the first glioma-related surgical procedure, to determine the overall survival.

## 3 Results

### 3.1 Symptom frequencies

**Figure 1:**
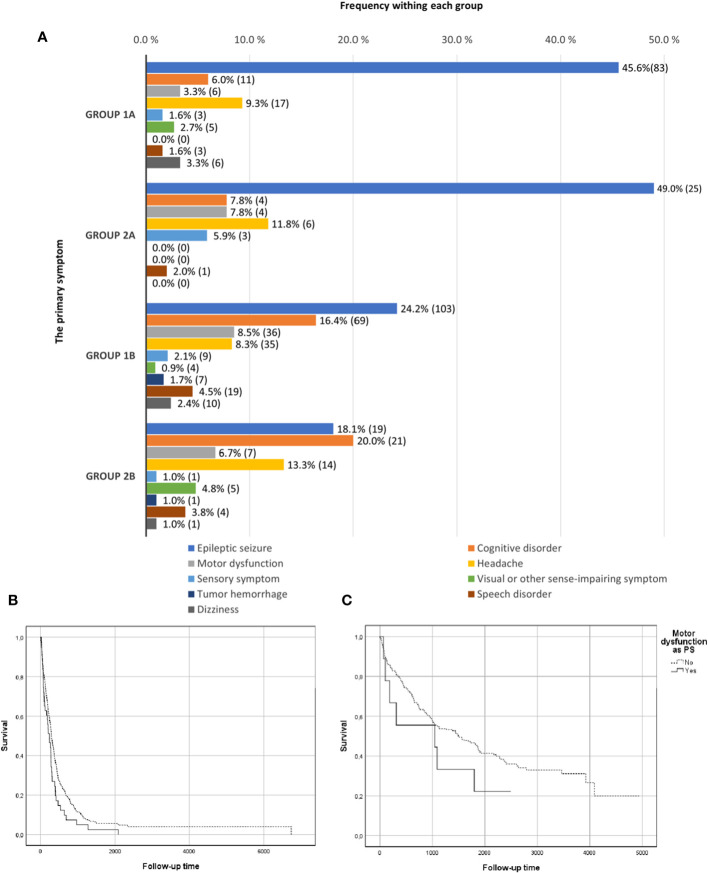
Primary symptom frequencies. **(A)** Primary symptom frequencies in groups 1 (retrospective) and 2 (prospective) divided by grade subgroups: 1A (grade II–III), 2A (grade II–III), 1B (grade IV), and 2B (grade IV). In addition, the survival curves for motor dysfunction in the combined retrospective and prospective groups. **(B)** Survival curves in kaplan-meier analysis for motor dysfunction as PS in grade 4 gliomas. **(C)** Survival curves in kaplan-meier analysis for motor dysfunction as PS in grade 2-3 gliomas.

**Figure 2:**
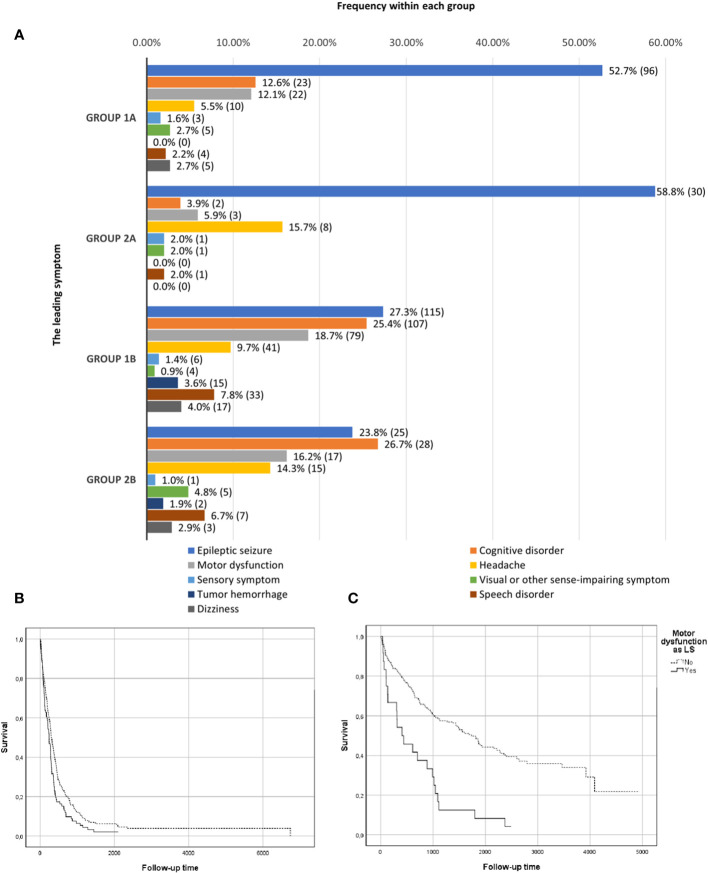
Leading symptom frequencies. **(A)** Leading symptom frequencies in groups 1 (retrospective) and 2 (prospective) divided by grade subgroups: 1A (grade II–III), 2A (grade II–III), 1B (grade IV), and 2B (grade IV). In addition, the survival curves for motor dysfunction in the combined retrospective and prospective groups. **(B)** Survival curves in kaplan-meier analysis for motor dysfunction as LS in grade 4 gliomas. **(C)** Survival curves in kaplan-meier analysis for motor dysfunction as LS in grade 2-3 gliomas.

Epileptic seizure was the most common PS and LS in both groups. PS frequencies for the subgroups are shown in (**Figure 1A**), and LS frequencies for the subgroups are shown in (**Figure 2A**). Cognitive disorder, headache, and motor dysfunction were the next most common symptoms among both groups. The distribution of symptoms was very similar between the subgroups. The PS could not be determined reliably for 190 patients (32%) in group 1 and 38 patients (24%) in group 2. The LS could not be determined for 16 patients (3%) in group 1 and five patients (3%) in group 2.

### 3.2 Molecular characteristics

In the retrospective data, seizure as the PS and the LS was associated with *IDH* mutation in the entire group 1 (p = 0.001 and p < 0.001 for PS and LS, respectively). In subgroup 1A, seizure was associated with *IDH* mutation, but only as the LS (p = 0.001). In subgroup 1B, no association was observed. In addition, motor dysfunction as the LS was associated with *IDH* wild-type tumors in the entire retrospective group and in subgroup 1A (p = 0.001 for each), but not when it was the PS.

The same tests were then studied in the prospective material to determine associations between *IDH* mutation and PS or LS. Seizure was associated with *IDH* mutation both as the PS and as the LS in the entire prospective group (p < 0.001 for each symptom comparison using the chi-square test). The same associations were observed separately in subgroup 2A (p = 0.023 for PS; p = 0.038 for LS) and subgroup 2B (p = 0.003 for PS; p = 0.017 for LS). Motor dysfunction showed similar associations in the prospective setting. Motor dysfunction as the LS was associated with wild-type *IDH* in the entire group and in subgroup 2A (p = 0.009 and p = 0.010, respectively). Unlike results in the retrospective group, cognitive disorder correlated with wild-type *IDH* in the entire prospective group (p = 0.053 for PS; p = 0.018 for LS).

Co-deletion of 1p19q and its relation to the symptoms were analyzed in the main groups. In the retrospective group, the absence of co-deletion was associated with motor dysfunction as the LS (p = 0.017). In the prospective group, cognitive disorder was associated with the absence of co-deletion as the PS and the LS (p = 0.010 and p = 0.023, respectively).

### 3.3 Symptoms and prognosis

To study the effect of symptoms on survival, we first assessed the results for univariate analysis in the retrospective data (group 1) for PS; results are presented in **Table 2**. In the entire group, epileptic seizure and a visual or other sense-impairing symptom as the PS were associated with a better prognosis, whereas motor dysfunction, cognitive disorder, and dizziness were associated with a worse outcome. In grade 2–3 diagnoses (subgroup 1A), only epileptic seizure was associated with a better outcome; in that group, dizziness and a sensory PS were associated with a worse outcome. In the grade 4 glioma group (subgroup 1B), epileptic seizure was associated with a better prognosis. Dizziness and motor dysfunction as the PS were associated with a worse outcome in grade 4 gliomas. Tumor hemorrhage, headache, and speech disorder as the PS had no significant prognostic value in the retrospective group.

**Table 2:** Prognostic value of the primary symptoms in patients with diffuse glioma in group 1 by log-rank test.

Primary Symptom	1A (G 2–3)			1B (G 4)			Whole Group (G 2–4)		
	Prognosis (p)^a^	95% CI for Mean Survival		Prognosis (p)^a^	95% CI for Mean Survival		Prognosis (p)^a^	95% CI for Mean Survival	
		Lower	Upper		Lower	Upper		Lower	Upper
**Epileptic seizure**	+ (0.002) [+/N.S.]	1570	2308	+ (0.034) [+/N.S.]	393	617	+ (<0.001) [+/N.S.]	967	1395
**Motor dysfunction**	N.S. [N.S./N.S.]	850	2185	– (0.026) [–/N.S.]	169	412	– (0.037) [N.S./N.S.]	275	657
**Cognitive disorder**	N.S. [N.S./–]	581	1710	N.S. [N.S./N.S.]	242	599	– (0.005) [–/–]	329	701
**Headache**	N.S. [N.S./N.S.]	577	1350	N.S. [N.S./N.S.]	349	729	N.S. [N.S./N.S.]	498	912
**Sensory symptom**	– (0.001) [N.S./–]	0	449	N.S. [N.S./N.S.]	196	841	N.S. [N.S./N.S.]	180	696
**Visual or other sense-impairing symptom**	N.S. [N.S./N.S.]	1043	3040	N.S. [N.S./N.S.]	0	1925	+ (0.019) [+/N.S.]	755	2378
**Tumor hemorrhage**	N/A			N.S. [–/N.S.]	113	449	N.S. [–/N.S.]	113	449
**Speech disorder**	N.S. [N.S./N.S.]	433	1150	N.S. [N.S./N.S.]	206	565	N.S. [N.S./N.S.]	275	606
**Dizziness**	– (0.020) [N.S./–]	4	788	– (0.021) [N.S./N.S.]	60	311	– (0.006) [N.S./–]	92	437

**+**Predicts a better prognosis compared with other patients. **–** Predicts a worse prognosis compared with other patients. G, tumor grade; N/A, not available; N.S., no significant prognostic value.

a Results reported separately for Tampere and Turku cohorts are shown individually in brackets.

The nine symptom categories were then assessed as the LS for their prognostic value in the retrospective study group; results are shown in **Table 3**. Epileptic seizure remained the only positive predictor in the entire group; prognosticators for worse outcome were motor dysfunction, cognitive disorder, tumor hemorrhage, and dizziness. For grade 2–3 diagnoses (subgroup 1A), seizure was the only predictor of better outcome, and motor dysfunction, cognitive disorder, and speech disorder were predictors of worse outcome. In grade 4 gliomas (subgroup 1B), seizure predicted better survival; conversely, motor dysfunction predicted worse survival. Headache or visual or other sense-impairing symptom as the LS did not have any significant prognostic value in the retrospective group.

**Table 3:** Prognostic value of the leading symptoms in patients with diffuse glioma in group 1 by log-rank test.

Leading Symptom	1A (G 2–3)			1B (G 4)			Whole Group (G 2–4)		
	Prognosis (p)^a^	95% CI for Mean Survival		Prognosis (p)^a^	95% CI for Mean Survival		Prognosis (p)^a^	95% CI for Mean Survival	
		Lower	Upper		Lower	Upper		Lower	Upper
**Epileptic seizure**	+ (<0.001) [N.S./N.S.]	1585	2245	+ (0.014) [+/N.S.]	411	644	+ (<0.001) [+/+]	996	1390
**Motor dysfunction**	– (0.003) [N.S./N.S.]	432	1027	– (0.032) [–/N.S.]	243	386	– (<0.001) [–/N.S.]	315	508
**Cognitive disorder**	– (0.004) [–/N.S.]	413	1075	N.S. [N.S./N.S.]	290	563	– (<0.001) [–/N.S.]	346	603
**Headache**	N.S. [N.S./N.S.]	599	2179	N.S. [N.S./N.S.]	336	668	N.S. [N.S./N.S.]	453	935
**Sensory symptom**	N.S. [N.S./N.S.]	1126	2065	N.S. [N.S./N.S.]	167	807	N.S. [N.S./N.S.]	433	1279
**Visual or other sense-impairing symptom**	N.S. [N.S./N.S.]	1158	3015	N.S. [N.S./N.S.]	277	389	N.S. [N.S./N.S.]	539	2076
**Tumor hemorrhage**	N/A			N.S. [N.S./N.S.]	38	589	– (0.003) [–/N.S.]	38	589
**Speech disorder**	– (0.044) [–/N.S.]	202	754	N.S. [N.S./N.S.]	123	1501	N.S. [–/N.S.]	157	1319
**Dizziness**	N.S. [N.S./N.S.]	66	954	N.S. [N.S./N.S.]	144	432	– (0.028) [N.S./N.S.]	186	491

**+**Predicts a better prognosis compared with other patients. **–** Predicts a worse prognosis compared with other patients. G, tumor grade; N/A, not available; N.S., no significant prognostic value.

a Results reported separately for Tampere and Turku cohorts are shown individually in brackets.

To validate the findings observed in the retrospective population, the associations were tested in the prospective cohort. First, the PS in the prospective study (group 2) were assessed (**Table 4**). As in the retrospective group, epileptic seizure predicted better outcomes, and motor dysfunction predicted worse outcomes, in the entire group. Motor dysfunction was the only predictive factor associated with poor survival in grade 2–3 disease (subgroup 2A). In grade 4 gliomas (subgroup 2B), cognitive disorder predicted worse survival; interestingly, headache was associated with better survival. The remaining PS had no significant prognostic value in the prospective group.

**Table 4:** Prognostic value of the primary symptoms in patients with diffuse glioma in group 2 by log-rank test.

Primary symptom	2A (G 2–3)			2B (G 4)			Whole Group (G 2–4)		
	Prognosis (p)	95% CI for Mean Survival		Prognosis (p)	95% CI for Mean Survival		Prognosis (p)	95% CI for Mean Survival	
		Lower	Upper		Lower	Upper		Lower	Upper
**Epileptic seizure**	N.S.	2253	4139	N.S.	521	1340	+ (0.002)	1530	3039
**Motor dysfunction**	– (<0.001)	9	309	N.S.	84	814	– (<0.001)	96	616
**Cognitive disorder**	N/A			– (0.006)	143	380	N.S.	318	968
**Headache**	N.S.	745	4957	+ (0.020)	687	2090	N.S.	1131	3339
**Sensory symptom**	N.S.	393	4105	N/A			N.S.	993	4062
**Visual or other sense-impairing symptom**	N/A			N.S.	0	1674	N.S.	0	1674
**Tumor hemorrhage**	N/A			N/A			N/A		
**Speech disorder**	N/A			N.S.	131	1700	N.S.	445	1653
**Dizziness**	N/A			N/A			N/A		

**+**Predicts a better prognosis compared with other patients. **–**Predicts a worse prognosis compared with other patients. G, tumor grade; N/A, not available; N.S., no significant prognostic value.

Last, the LS were evaluated in the prospective group; results are shown in **Table 5**. Epileptic seizure was the only predictor of better survival. Motor dysfunction, cognitive disorder, sensory symptom, and dizziness predicted poor survival in the entire group. Motor and sensory symptoms had prognostic value for poor survival in grade 2–3 disease (subgroup 2A). In grade 4 gliomas (subgroup 2B), dizziness correlated with poor survival and was the only symptom with prognostic significance. The remaining LS did not have any significant prognostic value in the prospective setting.

**Table 5:** Prognostic value of the leading symptoms in patients with diffuse glioma in group 2 by log-rank test.

Leading Symptom	2A (G 2–3)			2B (G 4)			Whole Group (G 2–4)		
	Prognosis (p)	95% CI for Mean Survival		Prognosis (p)	95% CI for Mean Survival		Prognosis (p)	95% CI for Mean Survival	
		Lower	Upper		Lower	Upper		Lower	Upper
**Epileptic seizure**	N.S.	2348	3818	N.S.	598	1213	+ (0.001)	1600	2791
**Motor dysfunction**	– (<0.001)	36	308	N.S.	156	837	– (<0.001)	150	714
**Cognitive disorder**	N/A			N.S.	232	647	– (0.011)	291	806
**Headache**	N.S.	2241	5676	N.S.	353	761	N.S.	574	3227
**Sensory symptom**	– (0.001)	24	24	N.S.	171	171	– (<0.001)	0	242
**Visual or other sense-impairing symptom**	N/A			N.S.	0	1674	N.S.	177	1917
**Tumor hemorrhage**	N/A			N/A			N/A		
**Speech disorder**	N/A			N.S.	302	1416	N.S.	463	1437
**Dizziness**	N/A			– (0.002)	46	293	– (<0.001)	46	293

**+**Predicts a better prognosis compared with other patients. **–**Predicts a worse prognosis compared with other patients. G, tumor grade; N/A, not available; N.S., no significant prognostic value.

Kaplan-Meier survival curves for motoric dysfunction as the PS in the whole combined study material (groups 1 and 2) are shown in **Figure 1B** (grade 4) and **Figure 1C** (grade 2-3). Furthermore, motoric symptom as LS is presented in **Figure 2B** (grade 4) and **Figure 2C** (grade 2-3).

The aforementioned results included few biopsied patients. Further analyses were conducted with these biopsied patients excluded. Group 1 has total of 41 biopsies (N_1A_ = 21 and N_1B_ = 20). Group 2 has 27 biopsied patients (N_2A_ = 14 and N_2B_ = 13). With these exclusions the statistically significant results largely remain, except the following: In the prospective group 2 grade 2-4 gliomas, motor disorder still showed clear trend of poor survival as PS and LS (p = 0.054 and p = 0.056, respectively). Additionally dizziness lost its predictive value as PS in subgroup 1A and as LS in grade 2-4 gliomas.

### 3.4 Other associations

In addition to the molecular characteristics described earlier, associations for sex, age, and tumor grade were tested. First, we assessed the associations in the retrospective population and the same analyses were then tested in the prospective group (**Table 6**). With regard to gender, men in group 1 seemed to have more epileptic seizures as the PS, and women in group 1 experienced more cognitive disorder as the LS. Women had headache more often than men as the LS in the prospective setting. Similarities between the groups were seen in the correlation between tumor grade and seizure or cognitive disorder. Seizure correlated with lower tumor grades, and cognitive disorder was associated with grade 4 tumors. Interestingly, in the retrospective group, motor dysfunction as the PS and tumor hemorrhage and speech disorder as the LS correlated with grade 4 tumors. Age associations were similar between groups 1 and 2. Seizure and headache correlated with younger age. Cognitive disorder correlated with older age; motor system correlated with older age as well, except as the PS in group 1.

**Table 6:** Symptom associations with gender, age, and grade of tumor in groups 1 and 2 respectively.

Group 1							
Symptom		Gender	p	Grade	p	Age	p
**Epileptic seizure**	**PS**	Male	0.060	II–III	<0.001	Lower age	<0.001
	**LS**			II–III	<0.001	Lower age	<0.001
**Motor dysfunction**	**PS**			IV	0.047		
	**LS**					Higher age	0.016
**Cognitive disorder**	**PS**			IV	0.002	Higher age	<0.001
	**LS**	Female	0.043	IV	0.001	Higher age	<0.001
**Headache**	**PS**					Lower age	0.003
	**LS**					Lower age	0.002
**Visual or other sense-impairing symptom**	**PS**					Lower age	0.016
**Tumor hemorrhage**	**LS**			IV	0.036		
**Speech disorder**	**LS**			IV	0.030		
Group 2							
Symptom		Gender	p	Grade	p	Age	p
**Epileptic seizure**	**PS**			Lower grade	<0.001	Lower age	<0.001
	**LS**			Lower grade	<0.001	Lower age	<0.001
**Motor dysfunction**	**PS**					Higher age	0.017
	**LS**					Higher age	0.002
**Cognitive disorder**	**PS**					Higher age	0.002
	**LS**			IV	0.003	Higher age	0.003
**Headache**	**PS**					Lower age	0.034
	**LS**	Female	0.008			Lower age	0.012

The chi-square test was used for gender and grade, and the Mann-Whitney U test was used for age association.

### 3.5 Multivariate analysis

Finally, the prognostic significance of different symptoms was evaluated in a Cox multivariate analysis for the entirety of pooled data, including both groups 1 and 2. In addition, odds ratios (ORs) for mortality were reported. Independent prognostic factors were *IDH* mutation (OR = 0.380; 95% CI, 0.263–0.550), older age (OR = 1.573; 95% CI, 1.350–1.834), high preoperative Karnofsky performance score (OR = 0.976; 95% CI, 0.971–0.982), high tumor grade (OR = 1.329; 95% CI, 1.110–1.592), and motor dysfunction as the PS (OR = 1.636; 95% CI, 1.147–2.332).

In a separate multivariate analysis for oligodendrogliomas, the forward stepwise regression model could not be completed. The low number of oligodendroglial tumors was the most likely reason for this result.

### 3.6 Updated WHO 2021 classification

Similar symptom trajectory was to be seen with the new WHO 2021 classification of gliomas in both groups 1 and 2. Epileptic seizure as PS remained as a positive prognosticator in *IDH* wildtype glioblastomas (p = 0.045 in Chi-squared test). Following, the motor symptom was an indicator of poor prognosis in *IDH* wildtype glioblastomas as PS (p = 0.019 in Chi-squared test) in group 1.

Motor symptom was an independent prognostic marker in COX multivariate analysis for poor survival in group 1 *IDH* wildtype glioblastomas. In this analysis were accounted the preoperative Karnofsky performance score and age.

## 4 Discussion

Epileptic seizure was the most frequent PS and LS of glioma. The second most common symptom was a cognitive disorder, followed by a motor dysfunction and headache. Excluding headache, all these symptoms were associated with prognosis. However, only motor dysfunction as the PS was independently associated with poor survival in a multivariate analysis (OR = 1.636; 95% CI, 1.147–2.332).

Most importantly, we demonstrated that motor dysfunction as a PS could be used as a predictor of poor prognosis in an independent manner, even after the 2021 WHO-defined molecular classification had been taken into account. This finding could be used as a personalized clinical tool in planning the primary operation strategy—whether to biopsy or make a resection. Our finding indicate that it might be better to biopsy a patient with HGG that is presenting with motor dysfunction. Although additional prospective research is needed to account the extent of resection with these patients. In addition, motor dysfunction status as a PS could play a role in designing additional oncological treatment modalities. In frontal or temporal gliomas, the symptom pattern could be used as a reference to consider supramarginal resection, which may lead to improved survival (24, 25). Surgical treatment targets aim to improve the survival of and the quality of life for patients with high-grade glioma. Maximum, safe resection of the tumor is associated with better survival in patients with grade 4 glioma without significant worsening of the neurological status (8). The extent of surgery, however, depends on the location of the tumor. For example, patients with gliomas that affect the motor pathway can be susceptible to postoperative paresis or plegia, which would worsen the quality of life (9). This potential for complications must be taken into account when evaluating the extent of resection. The treatment plan must be evaluated individually with every patient to achieve a longer prognosis but maintain the best possible neurological status.

Our data, like previously reported data, show that epileptic seizure as the PS correlates with better survival in patients with glioma patients (5, 15–17, 26, 27). The predictive nature of this symptom could be explained in part by the location or growth of the tumor and its molecular properties. Preoperative seizures have been linked to *IDH* mutation status in low-grade gliomas but not usually in grade 4 gliomas (6, 26, 28). Our study has similar results with seizure as either the PS or the LS. Interestingly, in the prospective setting, seizure was associated with *IDH* mutated astrocytomas in grade 4 gliomas as well. Seizure was not an independent prognostic factor, though *IDH* mutation was, and the correlation between these factors was clearly presented. The tumor growth rate, for one, could influence the epileptic properties of the tumor. One study suggested that a faster growth rate in temporal and insular gliomas led to seizures more often in low-grade gliomas, but fast-growing high-grade gliomas presented with other symptoms (29). Seizures commonly lead to faster imaging studies and therefore reduce the delay of diagnosis. Together, these properties of glioma-related epilepsy could explain the tumor biology behind the prognostic value. We showed that cognitive disorder was associated with worse survival as the PS and LS but not as an independent prognosticator. Earlier studies evaluating the LS have reflected similar results (18).

Our study included data from all patients who underwent operation for brain tumors within two geographically defined areas that cover approximately 2 million inhabitants, or nearly half the Finnish population. The two included university hospitals are the only tertiary care centers in their regions and are responsible for providing neurosurgical services. Thus, the study provides important epidemiological information about the symptom frequency in an unbiased manner. Our results on the symptom spectrum confirm the results of previous smaller studies: epileptic seizure is the most common symptom in all tumor groups (2, 7), followed by cognitive disorder, motor dysfunction, and headache. The prevalence of headache did not seem to be more common in our study than in the general population (30–32). Better survival of patients with headache might be explained with these gliomas being coincidental findings through a very common and unspecific symptom. This likely leads to faster interventions of the tumor and therefore better survival. In other comparisons, the symptoms reported in our study were similar to those reported previously (30, 33).

Our study had limitations with its retrospective properties. Patients can experience a broad spectrum of symptoms when suffering from a diffuse glioma. Symptoms can develop over time, and patients may not accurately recall the time spectrum of the symptoms, especially with cognitive symptoms. By including a prospective follow-up cohort, we more accurately defined the PS; however, it remained undefined for approximately one quarter of the study population. Otherwise, the results between groups 1 and 2 were very similar. In addition, a new WHO 2021 criteria for gliomas was published after this study was conducted. Therefore, we did not have all the required molecular data from all the patients before the new classification, e.g. TERT promoter, CDKN2A/B and chromosomes 7/10 status. We were unable to determine the MGMT status of the tumor samples, since it was not routinely assessed in our clinical practice, nor did we have the true extent of the resection reliably available for the whole study population. In grade 2 gliomas, there has most likely been an increase in favor for chemotherapy.

This study has its value as the clinician’s tool. Knowledge of the symptom trajectory and the value they serve as prognosticators in the first stages the tumor has been found, before any of the molecular information is available. In addition, this study has its strength reporting these results in large cohorts and in systematic manner. Low-income countries can lack the availability for the newer molecular analytics and therefore this could serve as valuable information regarding the glioma´s nature (34).

In conclusion, motor dysfunction as the PS in patients with glioma predicts worse survival and functions as an independent prognostic factor. We did not asses the combination of symptom burden regarding their effect on prognosis, though this would be an interesting topic for further studies. Additional prospective research is needed to validate the use of PS and LS as clinical prognosticator tools.

## Data availability statement

The original contributions presented in the study are included in the article/supplementary material. Further inquiries can be directed to the corresponding author.

## Ethics statement

The studies involving humans were approved by Ethical committee of the Pirkanmaa Hospital District. The studies were conducted in accordance with the local legislation and institutional requirements. The participants provided their written informed consent to participate in this study.

## Author contributions

TK: Conceptualization, Data curation, Formal analysis, Investigation, Software, Validation, Visualization, Writing – original draft, Writing – review & editing. JP: Conceptualization, Data curation, Investigation, Supervision, Validation, Writing – review & editing. JS: Conceptualization, Data curation, Investigation, Validation, Writing – review & editing. MiR: Conceptualization, Formal analysis, Validation, Writing – review & editing. JF: Conceptualization, Data curation, Investigation, Validation, Writing – review & editing. MG: Conceptualization, Data curation, Investigation, Validation, Writing – review & editing. MeR: Conceptualization, Data curation, Investigation, Validation, Writing – review & editing. KG: Conceptualization, Writing – review & editing. MN: Conceptualization, Writing – review & editing. VV: Conceptualization, Data curation, Investigation, Validation, Writing – review & editing. KN: Conceptualization, Formal analysis, Investigation, Supervision, Validation, Writing – review & editing. HH: Conceptualization, Data curation, Formal analysis, Investigation, Methodology, Supervision, Validation, Writing – review & editing. JH: Conceptualization, Data curation, Formal analysis, Investigation, Methodology, Project administration, Software, Supervision, Validation, Visualization, Writing – original draft, Writing – review & editing.
